# Intergenerational impacts of maternal mortality: Qualitative findings from rural Malawi

**DOI:** 10.1186/1742-4755-12-S1-S1

**Published:** 2015-05-06

**Authors:** Junior Bazile, Jonas Rigodon, Leslie Berman, Vanessa M Boulanger, Emily Maistrellis, Pilira Kausiwa, Alicia Ely Yamin

**Affiliations:** 1 Partners In Health /Abwenzi Pa Za Umoyo, Neno, Malawi; 2 Partners In Health / Inshuti Mu Buzima, Rwinkwavu, Rwanda; 3 Franςois-Xavier Bagnoud Center for Health and Human Rights, Harvard University, Boston, Massachusetts, USA; 4 Division of General Pediatrics, Boston Children’s Hospital, Boston, Massachusetts, USA; 5 Global Health and Population, Harvard School of Public Health, Boston, Massachusetts, USA

**Keywords:** Maternal and child health, infant survival, maternal mortality, health system, Malawi

## Abstract

**Background:**

Maternal mortality, although largely preventable, remains unacceptably high in developing countries such as Malawi and creates a number of intergenerational impacts. Few studies have investigated the far-reaching impacts of maternal death beyond infant survival. This study demonstrates the short- and long-term impacts of maternal death on children, families, and the community in order to raise awareness of the true costs of maternal mortality and poor maternal health care in Neno, a rural and remote district in Malawi.

**Methods:**

Qualitative in-depth interviews were conducted to assess the impact of maternal mortality on child, family, and community well-being. We conducted 20 key informant interviews, 20 stakeholder interviews, and six sex-stratified focus group discussions in the seven health centers that cover the district. Transcripts were translated, coded, and analyzed in NVivo 10.

**Results:**

Participants noted a number of far-reaching impacts on orphaned children, their new caretakers, and extended families following a maternal death. Female relatives typically took on caregiving responsibilities for orphaned children, regardless of the accompanying financial hardship and frequent lack of familial or governmental support. Maternal death exacerbated children’s vulnerabilities to long-term health and social impacts related to nutrition, education, employment, early partnership, pregnancy, and caretaking. Impacts were particularly salient for female children who were often forced to take on the majority of the household responsibilities. Participants cited a number of barriers to accessing quality child health care or support services, and many were unaware of programming available to assist them in raising orphaned children or how to access these services.

**Conclusions:**

In order to both reduce preventable maternal mortality and diminish the impacts on children, extended families, and communities, our findings highlight the importance of financing and implementing universal access to emergency obstetric and neonatal care, and contraception, as well as social protection programs, including among remote populations.

## Background

The population in Malawi was estimated at 16.4 million in 2013 [1], almost double the country’s population in 1987 [2]. Nearly half of the population lives below the poverty line, the majority of whom live in rural areas, where 90 per cent of households depend on rain-fed subsistence farming [2]. Forty-five per cent of the population is under 15 years of age, making it one of the youngest populations in the region [3-5]. While there has been a decrease in maternal mortality, from 910 per 100,000 live births in 1990 to 675 per 100,000 live births in 2010, the maternal death rate remains among the highest in the world [5,6]. A combination of high total fertility and limited access to contraception increases lifetime risk of maternal mortality [7] – the probability that a 15 year old woman will eventually die from a maternal cause - which for Malawi is estimated at one in thirty-six[6]. Modern contraceptive prevalence stood at 42% in 2010 [8], below the regional average of 58.1% in 2009 [9], access to contraception remains limited for adolescents and rural communities [3], and the total fertility rate is very high, at 5.7 children per woman in 2010 [3], with substantial differences between rural and urban areas (6.1 and 4.0 in rural and urban areas respectively).

Given that the majority of all maternal deaths are preventable [10], maternal mortality is a reflection both of the functioning of a health system and of the status of women in society. Malawi continues to face a number of challenges with respect to weak health infrastructure—offering inequitable access to and utilization of an essential package of health services—as well as significant health professional shortages, and low institutional capacity [11]. These factors are exacerbated in rural remote areas, where approximately 85% of the population resides [3].

The link between maternal death and infant survival is well established [12-14]. However, few studies [15-17] have attempted to capture the far-reaching impacts of maternal death on children and families, thereby discounting the importance of addressing this public health challenge, even where resources are limited. We conducted a multi-country study in select districts in four countries in sub-Saharan Africa, including Neno, a remote, rural district in southern Malawi, to provide qualitative insight on the intergenerational impacts of maternal mortality. Our results add significant new findings to an emerging body of evidence [13,15-18] on the far-reaching negative effects of maternal death on other children in the household, as well as on index children (children for whom the mother died during or shortly after their delivery), and identify linkages through which these impacts are related. Our paper offers a discussion of key qualitative findings in order to provide a context-specific overview of the impacts of maternal deaths on living children and extended family in Neno, Malawi, and offers recommendations for mitigating their far-reaching effects.

## Methods

### Program description

The data collected in Neno, Malawi forms one component of a four-country mixed methods study that was conducted in Tanzania, Ethiopia, Malawi, and South Africa on the impacts of maternal deaths on living children. The findings from other countries, as well as comparative analyses across countries, are or will be published elsewhere [15]. The research in Malawi was conducted by the Francois-Xavier Bagnoud Center for Health and Human Rights at Harvard University in partnership with Partners In Health (PIH), and its Malawian sister organization, Abwenzi Pa Za Umoyo (PIH-APZU).

### Population and setting

Neno District, with 137,078 inhabitants [19], is a rural, remote, and resource-limited area, with high HIV prevalence, a very young population, and very high fertility rates [19]. Neno was divided from Mwanza District in 2003 under the national decentralization program. Decentralization reforms in Malawi were introduced in response to perceived need for more focus on rural areas, where the majority of Malawians reside, in order to promote participatory poverty alleviation [20,21].

### Data collection

Data collection occurred between March 2013 and September 2013. Inclusion criteria for participation in the research study was defined as family members, community members, and stakeholders, 18 years old or older who have been affected by the death of a woman from maternal causes (the death of a woman during pregnancy, childbirth, or within 42 days of the termination of pregnancy, from any cause related to or aggravated by being pregnant), per the World Health Organization definition of a maternal death [22]. Participants were identified and recruited through partnerships with local health organizations, through community leaders who have integral knowledge of the families affected by maternal mortality in their communities, or through snow-ball sampling. Local research staff worked closely with health centers to identify and confirm that the women’s deaths were due to maternal causes through an examination of medical records.

Twenty key informant interviews were conducted with adult (≥18 years) guardians of orphaned children and adult family members of women who died due to maternal causes. Interview topics covered general characteristics of the family; the circumstances that led to the maternal death and the ensuing impacts on the children and family; and the availability and accessibility of services for maternal orphans. In the event of multiple maternal orphans in one family, the impacts of the mother’s death on each child were discussed. On average, the time interval between maternal death and interview with key informant was 75 months or 6 years and 3 months.

In addition to key informant interviews, 20 local stakeholder interviews were conducted with leaders of health organizations, community development officials, NGO leaders, school teachers, and government officials. Stakeholder interviews covered topics related to the availability of programs for vulnerable children and challenges for implementing programs and policies, including financial and political will.

Six sex-stratified focus groups were also conducted among 60 adult participants from the community including community leaders, community health promoters, community elders, teachers, family members of women who died of maternal causes, and other religious leaders. Focus group topics encompassed general community perceptions of orphans and vulnerable children, (including maternal orphans at different ages) such as infants and young children, school age children, and adolescents; community-level informal support for vulnerable children and families; and the availability of formal services and government programming for orphans.

All semi-structured in-depth interviews and focus groups were conducted by the research coordinator, with English-Chichewa translation, in a private location of the participant’s choice, and were digitally recorded. Each informant was personally invited to participate in our study and each in-depth interview and focus group took between 1.5 and 3 hours to complete. Key informants and focus group participants received 1,000 Malawian Kwacha (approximately 3 USD) for their participation. Informed consent was read verbatim by the research coordinator and all participants indicated consent through either a written signature or thumb print. Participant anonymity was protected in all reporting. Study protocols were approved by the Harvard School of Public Health Institutional Review Board and the National Health Sciences Research Committee in Malawi.

### Data analysis

All interviews and focus group discussions were transcribed from the digital recording and translated into English by the research coordinator. The transcripts were de-identified by the research team before any coding activity took place. Utilizing an inductive approach to coding, three research staff coded the transcripts, discussing and editing themes as they emerged, and developed a coding template. Key themes emerged including those related to familial decision-making regarding placement of children following a maternal death, extended family impact of maternal death, maternal and child health, barriers to receiving health care, the impact of maternal death on each child in the family and support available for orphaned children and families. The research team also looked into variables such as age at death, education, total number of births, and marital status of the women who died due to maternal causes. All analyses were conducted in NVivo 10.

## Results

Eighty five percent (85%) of the 20 stakeholders who participated in the study were male. About 55% of them had a University level of education, 25% and 20% had secondary level and primary level of education respectively. The mean age of the focus group discussion participants was 41 with a standard deviation of 15. About 88% of the focus group discussion participants were female. The demographic characteristics of the key informants are presented in Table 1. The key findings of our study are presented in the Table 2.

**Table 1:** Demographic characteristics of the key informants

N=20	Number	Per cent
Age		

18-27	7	35
28-37	4	20
38-47	1	5
48-57	4	20
58+	2	10
Don’t know	2	10

Sex		

Male Female	4 16	20 80

Education		

None	2	10
Primary	18	90

Marital Status		

Single	3	15
Living common law	5	25
Married Widowed	11 1	55 5

Number of children		

None	5	25
1-5	10	50
6-10	4	20
11+	1	5

**Table 2:** Summary of key findings.

Major Themes	Summary of Findings by Theme
**Caretaking by default after a maternal death**	- Female relatives from the maternal side were routinely called upon to care for orphaned children, often without having a choice in the matter. - Maternal death often exacerbated tensions between caregivers and extended family members who did not offer support for orphaned children. - Men frequently remarried before the mourning period ended, cutting ties with the maternal family.

**Barriers to accessing high quality care**	- The physical and economic challenges of accessing health centers played a role both in maternal death, and the provision of follow-up care to orphaned children. - Staffing shortages diminished the quality of care received by individuals who were able to access health centers. - Infants faced acute needs following maternal health. While health facilities provide free milk substitute for the first six months of life, these services were inconsistently available and difficult to access. - Older children faced health and nutritional risks related to protein deficiencies and low caloric intake.

**Financial hardships for caretakers and impacts on children**	- Caretakers faced economic hardship, stretching limited resources to support orphaned children. - Integrating orphaned children into a family often acted as a source of tension between spouses. - Families often turned to short-term, informal labor, to absorb the immediate impacts of caring for orphaned children, which can limit a family's opportunities for financial stability and independence. - Orphaned children were often called upon to take on additional household responsibilities, with preference showed towards biological children in allocation of expenses related to school and nutrition.

**Loss of childhood for orphans, especially female orphans**	- Orphaned children faced disadvantages related to educational opportunity, when families could not afford school fees and supplies. - Girl children were often expected to take on caretaking and household responsibilities, and faced pressures to find a partner at an early age in order to alleviate financial pressures on the family. - Losing a mother also had informational, emotional, and social costs for girl children.

**Government assistance and other support programs**	- Many participants did not know about support available through government institutions and NGOs. - Those who did seek support, often felt it was ineffective, non-transparent or difficult to access.

### Caretaking by default after a maternal death

Interviews and focus group discussions revealed that, unlike in the patrilineal North [23], the deceased woman’s side of the family typically convenes a meeting following a maternal death in Southern Malawi, to determine who will assume responsibility for the orphaned children, the family and community support available, and to evaluate the husband’s engagement in both caretaking and support. Women from the maternal side are virtually always called upon to become the primary guardian of orphaned children. In fact, grandmothers and aunts expressed that they had no choice but to accept this responsibility in the absence of anyone else who would come forward to care for the child. As one grandmother said:

It was a very big responsibility, and every day when I looked at the burden that I had upon me, I used to think that we should have exchanged so the one who died would be me. I would have loved my daughter to be alive to take care of the child and let me die instead. Because what was happening was that when the child starts crying, I could also cry and even eating was not easy. Unfortunately it so happened to be the time when we were completely blocked and going through financial hardships. I could even think or wonder if there was God in heaven, so that he could have exchanged the incident [let me die instead of my daughter], because I had stopped breastfeeding a long time ago so I could not breastfeed the child.

While some immediate family members attempted to support new caretakers by providing food and other resources, nearly one third of participants discussed how a maternal death could serve to exacerbate tensions between caregivers and fathers and extended family members who did not offer to assist in caretaking or to provide other material supports. Further, participants mentioned that when fathers and extended family members did pledge support, few followed through on such commitments:

The communities are supposed to join hands and facilitate contributions such as soap, clothes, body lotion and any other things that they can afford, but in practice what happens is that people do not help and tend to leave everything in the hands of the actual guardians.

Traditional custom in Southern Malawi dictates that, following a woman’s death, a husband is expected to contribute food, money, and other goods to the wife’s funeral, and to stay with the maternal family of the deceased woman for at least three months to provide for the children before making any significant life decisions, such as remarrying. However, participants mentioned that husbands often did not adhere to these responsibilities and finished mourning their wives before the three months had elapsed. As one key informant stated, the care of children is impacted significantly when fathers evade customary responsibilities:

These men don’t face any problems, they may just be sorrowful on that particular day on which they have lost their wives, but just after a few weeks they leave home and start looking around to remarry. Once they are married you will see they reduce the amount of care they provide, and even when you decide to follow him [a man who has recently lost his wife] you will discover that there is another lady who possibly will not take care of these children or might ill-treat them.

Key informants explained that, following the three-month mourning period at the mother-in-law’s compound (and sometimes prior to the conclusion of the mourning period), many men remarry and often cut or diminish ties with the maternal family, offering only minimal material support for the orphaned biological children, and visiting infrequently. As one guardian, a grandmother, recalled:

In my case, my child died in 1997 and left an infant and the father bought Lactogen [formula] and other things for the baby but after the funeral ceremony, the father disappeared for good until now.

Additionally, our study found no social sanctions imposed by traditional chiefs or community elders, nor legal ramifications for men failing to comply with their responsibilities toward their deceased wife and children. One key informant voiced frustration with the lack of a supportive legal system that could hold men accountable:

What I can say is marriages should be legally bound, so that, when this misfortune happens [maternal death], the government can easily intervene and require the man to be responsible for his children. What happens sometimes is that because there are no proper marriage foundations and procedures, once the wife dies, the husband will never be seen again and cannot be traced.

In our study population, no children were sent to live with their biological father once he remarried, and even when fathers did not remarry, the children were primarily taken care of by maternal family members. After remarriage, children from first wives were lower in priority for the biological father in terms of intra-household food distribution and other care. This could be due both to conditions of extreme poverty that limited the ability of biological fathers to support multiple households and a need to prove allegiance to the second wife and her family. Moreover, participants mentioned the notion that a stepmother would withhold equal treatment and affection, and as a result, maternal family members choose to keep orphans under their own care rather than send them to live with their fathers. As one participant stated:

The other problem is when this man decides to take these [his biological] children we always fear that they [the children] will not be treated well by the stepmother, as it can be seen that many are women who are not able to take care of a child earnestly who are not their own. This compels relatives of the children to not allow the children to be taken by the father, rather they keep the children staying with them, and share with the children the little [resources] that they are able to find.

### Barriers to accessing high quality care

Registration at the health facility of a maternal death and the health status of the orphaned infant is critical to ensuring that children are enrolled into appropriate programs, including nutritional and other support services that are provided through health facilities. However, the remoteness, physical (i.e. treacherous roads), and economic difficulty of accessing health facilities, and the lack of available transportation, play a role not only in maternal deaths, but also as obstacles to the registration of vital events and to the provision of follow-up care to orphaned children. Particularly when a mother dies at home or en route to a health facility, the likelihood is substantially reduced that the death and status of the infant will be recorded at the health facility.

Participants commented that the most immediate impacts of maternal death are often felt by surviving infants who are in need of breast milk. Many expressed concern that index children were small for their age and were not getting appropriate nutrition and care. According to one key informant:

[I am] concerned because our child who was left behind is very young, we are unable to get basic needs for the baby…so that the child may grow as normally as any child who has both parents can grow...

Under national policy, health facilities are supposed to provide milk substitute for the first three to six months of life free of charge, and flour for porridge after six months. Nevertheless, in practice, our study results reveal that these critical nutritional supplements were available inconsistently. Many participants felt that the amount and duration of support were insufficient and that weekly visits to the health facility added an additional economic and practical burden to taking care of the orphan(s).

While these concerns were most acute for the youngest children, older children faced other health and nutritional risks related to lack of caloric consumption and protein. When asked about the health of older orphaned children in the community, this stakeholder said:

There will be problems in terms of development because as I have already said that food is insufficient for their bodies, and it is not nutritious food because there is no way you can just be feeding a baby on pumpkin leaves daily without anything else. [If you do so], the body will not develop and you will see that these children’s bodies are swollen, they are sick, and lacking a balanced diet.

And this stakeholder added that orphaned children are particularly vulnerable to infection:

Most of these children, simply for the reason that they had a bad background nutritionally, they look sickly. Most of the time when there is an outbreak, just like we had an outbreak of chicken pox (Varicella), these are the children [maternal orphans] who become more vulnerable because of low immunity.

At health facilities, families face additional barriers including poor quality of care and critical shortages of health workers. Despite government efforts to strengthen human resources for health, only 13% of health centers in 2008 had the requisite numbers of skilled health staff [4], and attracting staff to work in remote and rural areas has remained a severe problem in Malawi, despite government and PIH-led incentive programs. Our study highlighted staffing shortages as a key barrier to receiving quality care, for both women and children, noting that staff were too overburdened to provide sufficient information, counseling, and follow-up to mothers attending health facilities for antenatal and delivery care. One quarter of family members interviewed described labor and delivery experiences that involved referrals between multiple facilities that were supposed to, but ultimately did not, have the requisite staff or expertise to care for the laboring woman. Family members also cited a shortage of nursing staff at facilities, causing women to deliver their babies alone, and limiting access to care for orphans at the community level:

… for instance at this hospital we only have 8 nurses; only 8 nurses, and we have 3 wards, and these nurses have to rotate working day and night, so the same nurses have to go to the community. We don’t have a community health nurse, and this community health nurse is supposed to do such work in the community…

### Financial hardships for caretakers and impacts on children

While it was customary for female family and community members to take on caregiving responsibilities following a maternal death, we found that many already had children of their own, and were therefore forced to further stretch household resources to meet the additional needs of orphans:

In the first place, it should be mentioned that these children land in needy families who are already struggling to earn a living, so normally there is an over stretched household budget because there are some additional items that will be sought specifically for the orphaned children. As such, the families will intensify [the time they devote to] Ganyu activities [informal, small-task labor], which do not suffice to fill the needs for the household.

As this stakeholder explained, in an attempt to absorb the immediate financial shock of taking in an orphan, families often turn to short-term labor, or *ganyu*, which includes weeding or other forms of field labor, as an immediate source of income. However, *ganyu* has been found to conflict with one’s ability to devote time to independent labor, such as maintaining one’s own farm, which has the potential to reap greater benefits long-term, but for which the financial payoff is less immediate. Further, the stigma attached to *ganyu*, which is seen as a sign that a family has run out of food, can have negative social implications for a family within the village [24]. Moreover, despite the high proportion of female-headed households in Malawi, women are paid less than men for *ganyu*, and have fewer hours to devote to any form of income generating labor, due to caregiving responsibilities [24,25], substantially diminishing the overall return. As one female caregiver, a grandmother taking care of her daughter’s orphaned child, described:

You can see my situation, I can no longer go to farm in the gardens to get some money. You can see that I can no longer farm in my gardens because I have to pay close attention to the child. There is nothing that I can do to support myself apart from getting the milk from the hospital and that’s all.

Furthermore, female relatives who became new caretakers following a maternal death stated that integrating children into their families could be a source of tension with their husbands, contributing to conflict within the home, further limiting their financial security and stability. As one key informant recalled:

So I was left with the other children and other relatives promised to assist, but they never came back and because I was taking care of my relatives my husband was having difficulties with that and we separated.

As described above, the additional burden on caregivers often translated to a burden on children to help pick up the slack. Where resources were limited, stakeholders noted that non-orphaned children would be given preference for expenses related to school and nutrition, with orphaned children often fed separately, after non-orphan children had eaten, or not at all if food was scarce. Orphaned children were also required to take on additional household chores and labor in the fields, which could prohibit them from attending school. As one focus group participant said:

There is remarkable [school] absenteeism [among orphan children] due to lack of basic needs such as soap. Others give priority to their children and not the orphans, as a result there is a lot of [school] absenteeism in these children as compared to the other children in normal families. Other guardians will even buy clothes but just for their children…[while the orphaned child is] sent to do household chores such as drawing water, cooking etc. As a result, the child is never free to go to school.

### Loss of childhood for orphans, especially female orphans

Surviving children face numerous disadvantages as new caretakers struggle to integrate these children into their households. As explained by one participant:

Surviving children are given a lot of work to do which is beyond their age. Some may be abused sexually by their guardians just to get support. This may lead them to drop out of school and for girls they end up in early marriages and early pregnancies which may also end up maternal death.

While primary school is free in Malawi, fees must be paid in order to attend secondary school. Even if a family is able to pay the required fee, participants commented on the additional fees associated with school supplies and appropriate clothing, and the discrepancy in allocation of resources for these materials between orphans and non-orphans.

… children that still have their parents have very high chances of completing school [compared to] orphans. Guardians may easily sacrifice even their livestock for [their biological] child as compared to an orphan. Priority is given to their children as compared to orphans in cases of school fees, clothing as well as food. It is almost impossible for these guardians to sacrifice something for the orphans.

Boys are also seriously affected by maternal morbidity and mortality. As this orphan who was 16 when his mother passed away and 22 at the time of the interview stated:

I stopped going to school to take care of my mother while she was sick and she became pregnant and died after giving birth. Things have been different after her death. As of now I have the burden of taking care of that child.

Participants mentioned that girl children may be expected to take on caretaking and other household responsibilities, often caring for their orphaned younger siblings, making it difficult for them to continue attending school. As one stakeholder stated, “Many girls drop out of school because they are overburdened by household chores.” Participants also noted that girls often face family and social pressure to find a partner, as a means of relieving perceived burden on the household, thereby perpetuating a cycle of early marriage, heightened risk for maternal mortality associated with early childbearing [26], and limited future opportunity. As one focus group participant mentioned, this pressure seems to disproportionately affect female orphans compared to biological girl children:

Some guardians, once they have seen that the child is of age, force them into early marriages instead of encouraging them to go to school so that they will be relieved of their responsibility [to care for the girl child] and they will make sure that their [biological] children go ahead with school…

Losing a mother does not just have practical implications for girl children relating to household economics, there are informational, emotional and social costs as well. When a mother dies her daughter loses a vital source of information about gender roles, relationships, behavior, the body and reproductive health. Several participants spoke about the encouraging and supportive nature of mothers, and the importance of maternal guidance for children’s motivation and success. Maternal orphanhood could have negative health implications for girl children, such as early sexual debut and pregnancy [27]. As one focus group participant described, the mother is responsible for ensuring that children adopt appropriate values and behavior, and additionally, the mother has specific responsibilities to set a positive social example for girl children, something which fathers cannot provide:

I am seeing something here, when a mother is alive she controls proper dressing for a girl child as well as boy child and instills in them cultural norms and values and they become well behaved. As a family, a man and a woman advise their children according to what is expected of a girl and a boy, both by the mother, and what is expected of a boy by the father, but I as a man cannot go and advise my daughter on what is [appropriate] for women. This would rarely happen in a single parent situation and would [negatively] affect the girl child.

### Government assistance and other support programs

Many participants did not know about the different forms of support that were available to them through governmental institutions and NGOs, and the processes for accessing such support. When participants did seek support, or were aware of such programs, they stated that the programs were often ineffective, non-transparent, or difficult to access. Challenges receiving support from NGOs and government programs included delays in receiving services once selected as a program recipient and difficulties returning to the health facility or other distribution sites to receive regular services and supplies. As one key informant explained:

Yes, the problem [with the government welfare program] was that…they just wrote down our names…They just wrote our names and then they kept quiet, they did not assist us. They came twice just to write our names and then we stopped them because we didn’t see any impact from their program.

Moreover, a focus group participant expressed frustration at the situation of her sister’s orphaned child, for whom formal assistance had been sought out, but was never received:

There are other organizations that take care of orphans but none of them accept him to be enrolled in their centers, saying that he is too old. As such, I just engage the child in piece works [ganyu] as a herd boy, or organize some firewood for him to sell, so I feel sorry for the child.

## Discussion

Our results add to an emerging body of evidence on the impacts of a maternal death on children, extended families and communities, as well as the mechanisms through which vulnerabilities emerge and are perpetuated following maternal death [15,17,18]. The findings from this study document the far-reaching effects that maternal mortality can have on child health and well-being, highlighting the impact of intersecting discriminations and disadvantages, such as gender inequality and poverty, in altering the trajectory of a child’s short- and long-term health and development outcomes.

Our findings revealed that maternal death serves as a catalyst for vulnerabilities specific to orphaned children related to health, nutrition, education, employment, early partnership, pregnancy, and household and caretaking responsibility. We found that fathers are, in the majority, relatively uninvolved in the care of their children following a maternal death, particularly among those who have remarried. Women disproportionately bear household burdens associated with taking in additional children and are often forced to take on paid labor alongside their usual caregiving responsibilities.

Gender inequalities were exacerbated both in the expectation that women would become caregivers of orphaned children while men were left to remarry, as well as in the related effects of this distribution of responsibility in women’s ability to access financial and material resources, including paid labor.

Following maternal deaths, orphaned children, and particularly the index child and other young children in our sample, are at increased risk of health complications and decreased likelihood of receiving appropriate nutrition, as well as education and other social development opportunities. We found that girl orphans in particular face high risks of school dropout, early marriage and early childbearing which, compounded with high birth rates and lower household wealth, among other factors, increases risk of maternal mortality [26,28] and perpetuates a cycle of poverty for the children who are left behind—girls who become pregnant before age 15 are five times more likely to die of maternal causes than women who are pregnant over the age of 20 [26,28]. Further, in our sample, 30% of the women who died of maternal causes had given birth to five or more children. In this context of high fertility coupled with high unmet need for contraception, both of which are driven by gender inequalities and weak health systems, women faced a greater risk of maternal mortality, thereby increasing the risk that children will be orphaned.

These results should be interpreted with limitations in mind. While this study illuminates the intergenerational impacts of maternal death on children in Neno, Malawi, our findings are not meant to be generalized beyond the study population. As referenced above, we conducted six sex-stratified focus groups in an effort to create a comfortable environment for participants to express themselves, and remove gender-based power differentials, which are particularly salient in Malawian culture. Focus group facilitators conscientiously engaged all participants, and utilized neutral open ended questioning techniques in an attempt to minimize response bias and mitigate additional socially-based power differentials amongst participants; yet we were unable to fully control for recall bias or the effect of any bias towards responses deemed to be more socially acceptable within groups. Our study population included only adult participants with a minimum age of 18 years. There were only two adult orphans that we were able to include as key informants in our study population. Stepmothers (the wife of fathers who got remarried after the death of their child’s mother) were not captured in our study population because as a custom in Malawi, children almost always stay with a maternal family member following a maternal death. This combination of factors resulted in an underrepresentation of orphans, men, and stepmothers in our study and the perspectives of these groups may not be depicted accurately in our findings.

## Conclusions

Malawi is a resource-limited country, and Neno a very remote, extremely poor area, in which the health system is inadequately supplied to respond to the needs of the population. The government of Malawi has emphasized health system strengthening in its Health Sector Strategic Plan (HSSP), particularly through increasing the number of skilled health workers. Despite these efforts, attracting staff to work in remote, rural areas has proven challenging. While the number of professional health care workers increased nationally by 53% from 5,453 in 2004 to 8,369 in 2010 [29], in rural settings such as Neno, there are significant challenges to retaining qualified health professionals as access to basic needs and transportation is extremely limited, infrastructure is poor, and there are frequent stock-outs making health care practice difficult.

Achieving Millennium Development Goal 5, reducing maternal mortality and providing universal access to reproductive health, will not be met without significant investment in strengthening health systems, with an emphasis on integrated sexual and reproductive health and rights, including providing accessible, quality Emergency Obstetric and Neonatal Care (EmONC), as well as contraceptive options, for all pregnant women, including those residing in remote areas [29].

The findings of our study are similar to those of other researchers looking at the impact of maternal death on living children [12,13,15], and add insight into the far-reaching and intergenerational impacts of maternal mortality, particularly in the context of weak health systems and gender norms that favor women as caretakers while limiting their economic independence in caring for their households. When one woman dies the lives of her children are deeply affected. Maternal mortality limits these children’s future opportunities as many either drop out of school to assume care-taking responsibilities for their household, or become absorbed into existing households which may not have the capacity to prioritize their health, nutrition, and education, largely due to economic constraints. These paths contribute to the perpetuation of low levels of education, lack of economic opportunities, cycle of early marriages, and heightened vulnerability to maternal deaths among orphaned girl children. Moreover, the economic impact of a maternal death spans not only generations of maternal orphans, it spills over into the lives of families who attempt to provide support for these orphans as well.

In order to truly improve outcomes for the most marginalized of Malawian children, comprehensive social protection at the government, district, and community levels are needed. However, this intermediary solution cannot replace the need to protect women’s right to life and promote gender equality by ending the cycle of preventable maternal mortality through concerted health systems strengthening that ensures universal access to sexual and reproductive health and rights, including the universal provision of family planning and EmONC, particularly in remote rural areas. At the community level, Non-Governmental Organizations, in collaboration with community members, including the Traditional Authorities, should provide education to families on the importance of women’s and children’s health and rights, maternal health, planning pregnancies, saving for birth, the importance of not delaying care, danger signs in pregnancy, breastfeeding, and appropriate care for children. They should also address social and cultural norms that impact women’s risks for maternal death, as well as the care of maternal orphans and other vulnerable children, and the role of fathers.

## Competing interests

The authors of this paper have no conflicts of interest to report.

## Authors’ contributions

Conceived and designed the study: AEY. Performed data collection: JB, PK. Analyzed the data: JB, LB, VMB. Wrote the paper: JB, LB, VMB, EM, JR, AEY. All authors read and approved the final manuscript.

## Peer review

Reviewer reports for this article can be found in Additional file 1.

## Supplementary Material

Additional file 1 Click here for file

## Declarations

This article has been published as part of *Reproductive Health* Volume 12 Supplement 1, 2015: True costs of maternal death. The full contents of the supplement are available online at http://www.reproductive-health-journal.com/supplements/12/S1. Publication charges for this supplement were funded by Family Care International and the FXB Center for Health and Human Rights.
