# Supplementary material for: Intergenerational impacts of maternal mortality: Qualitative findings from rural Malawi
Source: Reprod Health. 2015 May 6;12(Suppl 1):S1. doi: 10.1186/1742-4755-12-S1-S1 (PMC4423580; doi:10.1186/1742-4755-12-S1-S1)
Supplement: Additional file 1 [file 1742-4755-12-S1-S1-S1.pdf]

Referee's comments to the authors– this sheet WILL be seen by the author(s) and published with the article

Title	Intergenerational impacts of maternal mortality: Qualitative findings from rural Malawi
Author(s)	Junior Bazile, Jonas Rigodon, Leslie Berman, Vanessa M Boulanger, Emily Maistrellis, Pilira Kausiwa and Alicia E Yamin
Referee's name	Eric Chamot

When assessing the work, please consider the following points, where applicable:

1. Is the question posed by the authors new and well defined?
2. Are the methods appropriate and well described, and are sufficient details provided to replicate the work?
3. Are the data sound and well controlled?
4. Does the manuscript adhere to the relevant standards for reporting and data deposition?
5. Are the discussion and conclusions well balanced and adequately supported by the data?
6. Do the title and abstract accurately convey what has been found?
7. Is the writing acceptable?

Please make your report as constructive and detailed as possible in your comments so that authors have the opportunity to overcome any serious deficiencies that you find and please also divide your comments into the following categories:

- Major Compulsory Revisions (which the author must respond to before a decision on publication can be reached)
- Minor Essential Revisions (such as missing labels on figures, or the wrong use of a term, which the author can be trusted to correct)
- Discretionary Revisions (which are recommendations for improvement but which the author can choose to ignore)

Where possible please supply references to substantiate your comments.

When referring to the manuscript please provide specific page and paragraph citations where appropriate.

General comments:

The aim of this qualitative study was to assess the long-term impacts of maternal mortality in rural Malawi on orphan children, their caretakers, their family, and the community at large. The manuscript is generally well organized and well written; it addresses an under-researched topic of extremely high public health importance, in particular as it relates to United Nations Millennium Development Goals.

Major compulsory revisions:

There are four categories of actors who appear to be underrepresented in study data: orphans, men in general, fathers, and the wife of fathers who got remarried after the death of their child's mother (i.e., stepmothers). A lot is said about these actors in the paper, but very little space is dedicated to what they have to say personally about the issues raised by other actors (most/all of the quotes seem to be from female caretakers). If no useable/useful information was collected directly from orphans, men, fathers and women married to a man who has an orphan child, this should be stated in the limitation section of the discussion. Furthermore, the potential consequences of this underrepresentation on study findings should be discussed.

A related issues is that of orphan boys. Very little is said about them compared to orphan girls.

Information should be provided on local inheritance practices and how they influence the impacts of

Continued:

maternal mortality on the different actors in the community.

To facilitate reading, the characteristics and composition of the study sample should be summarized in a table. Similarly, key findings should be summarized in another table.

The only solutions proposed to address the challenges associated with maternal mortality are governmental solutions. What about community-based participatory solutions?

Minor essential revisions:

Data collection section, first line of the first paragraph. This is a detail, but formally “death of a woman during pregnancy, childbirth, or within 42 days of the termination of pregnancy is not an “inclusion criterion”. The authors did not recruit “deceased mothers”, but persons affected by the death of a mother. Furthermore, and if possible, information should be provided about the time interval between maternal death and interview with key informants (or, in other words, the age of the child who lost his mother).

Discretionary revisions:

None.

Referee's comments to the authors– this sheet WILL be seen by the author(s) and published with the article

Title	Intergenerational impacts of maternal mortality: Qualitative findings from rural Malawi
Author(s)	Junior Bazile, Jonas Rigodon, Leslie Berman, Vanessa M Boulanger, Emily Maistrellis, Pilira Kausiwa and Alicia E Yamin
Referee's name	Martha Wingate

When assessing the work, please consider the following points, where applicable:

1. Is the question posed by the authors new and well defined?
2. Are the methods appropriate and well described, and are sufficient details provided to replicate the work?
3. Are the data sound and well controlled?
4. Does the manuscript adhere to the relevant standards for reporting and data deposition?
5. Are the discussion and conclusions well balanced and adequately supported by the data?
6. Do the title and abstract accurately convey what has been found?
7. Is the writing acceptable?

Please make your report as constructive and detailed as possible in your comments so that authors have the opportunity to overcome any serious deficiencies that you find and please also divide your comments into the following categories:

- Major Compulsory Revisions (which the author must respond to before a decision on publication can be reached)
- Minor Essential Revisions (such as missing labels on figures, or the wrong use of a term, which the author can be trusted to correct)
- Discretionary Revisions (which are recommendations for improvement but which the author can choose to ignore)

Where possible please supply references to substantiate your comments.

When referring to the manuscript please provide specific page and paragraph citations where appropriate.

General comments: This manuscript is very well-written and informative. Thank you for the opportunity to review it.

Major compulsory revisions:

Minor essential revisions:
On line 64, please provide the denominator of the one in thirty-six. Is this pregnant women? Women of childbearing age?

In the data collection section, on line 107, the term "termination of pregnancy" is used. One can assume that this includes delivery of child, but is the term only related to live births or does it include spontaneous terminations (abortions, stillbirths, etc.)?

Discretionary revisions:
Throughout the paper the words "per cent" is seen, but typically it is seen as "percent". Also, please be consistent, per journal reviews , with the use of % or percent.

Comments from the Supplement Editor

1. Compulsory Revisions: Limitations: the limitations section is not adequate as written, one of the reviewers comments on the compulsory revisions in terms of sorting out who is who among the respondents, it seems as if men and consequences for male children are under represented, this should be addressed. In terms of the limitations of response bias and social acceptability bias, this also needs to be addressed. For example, one possible limitation to the findings is the heterogeneity of the focus groups. While the authors state that there were six sex-stratified focus groups, there is no mention of other power and social differentials in the participants that may have given more "voice" to some participants over others. How were these differences handled, what effect might these differentials had on discussion?

2. Compulsory Revisions: table is necessary to determine demographics of participants across the 3 categories of respondents, individual one to one interviews with "key informants, " stakeholders, and the focus group participants

3. Compulsory Revision: There is no distinguishing discussion about the differences in key informants and focus group participants, there is no discussion of the effects of the interactions of participants in the focus groups.

Minor Essential
1. Use of the verb "shared", this is used at least 7 times, it is a very idiomatic, Western term that might not be understood by readers from all countries, please replace with "told" or "said" or another verb
2. grammar: p. 6, line 114, change order of wording to adult (>18 years) guardians
3. p. 11: line 238: change verb from plays (singular(to "play" plural an change the wording from "but also in ensuring" to " but also as obstacles to registration of vital events…"
4. p 12: lines 276-282: the information here is on staffing shortages affecting mothers from obtaining care during labor, but the article's focus is supposed to be on barriers to children receiving health care and impacts of maternal mortality, how do these shortages affect the children's obtaining health care?
5. p 14 line 316, the word "Female" should not be capitalized
6. p. 14, lines 323-325, delete these lines, unnecessary for the quote as supporting the concept
7. p. 18, line 428: in women's ability to access **to** financial and material…" delete second "to"
8. p. 18, line 431, delete "and" at the beginning of the sentence
9. p. 18, line 438, delete second period after citation
